# Fast volumetric ultrasound facilitates high-resolution 3D mapping of tissue compartments

**DOI:** 10.1126/sciadv.adg8176

**Published:** 2023-05-31

**Authors:** Eun-Yeong Park, Xiran Cai, Josquin Foiret, Hanna Bendjador, Dongwoon Hyun, Brett Z. Fite, Robert Wodnicki, Jeremy J. Dahl, Robert D. Boutin, Katherine W. Ferrara

**Affiliations:** ^1^ Department of Radiology, Stanford University, Stanford, CA 94305, USA.; ^2^ Department of Biomedical Engineering, University of Southern California, Los Angeles, CA 90089, USA.

## Abstract

Volumetric ultrasound imaging has the potential for operator-independent acquisition and enhanced field of view. Panoramic acquisition has many applications across ultrasound; spanning musculoskeletal, liver, breast, and pediatric imaging; and image-guided therapy. Challenges in high-resolution human imaging, such as subtle motion and the presence of bone or gas, have limited such acquisition. These issues can be addressed with a large transducer aperture and fast acquisition and processing. Programmable, ultrafast ultrasound scanners with a high channel count provide an unprecedented opportunity to optimize volumetric acquisition. In this work, we implement nonlinear processing and develop distributed beamformation to achieve fast acquisition over a 47-centimeter aperture. As a result, we achieve a 50-micrometer −6-decibel point spread function at 5 megahertz and resolve in-plane targets. A large volume scan of a human limb is completed in a few seconds, and in a 2-millimeter dorsal vein, the image intensity difference between the vessel center and surrounding tissue was ~50 decibels, facilitating three-dimensional reconstruction of the vasculature.

## Teaser

Human-scale volumetric ultrasound imaging is accomplished in seconds using a large transducer aperture and distributed processing.

## INTRODUCTION

Ultrasound (US) imaging is used throughout medicine; however, operator-dependent acquisition and poor spatial resolution have limited the utility. Conventional US imaging uses a small linear or two-dimensional (2D) matrix probe to transmit (Tx) and receive (Rx) US signals. Typical limitations of conventional US imaging include small field-of-view, limited penetration compared to the size of the imaged object and diffraction-limited resolution. The integration of large numbers of transducer elements and signal acquisition channels has the potential to solve classical, important problems. The recent development of high–channel count ultrafast US systems offers the opportunity to capture images at a high frame rate using plane waves (PWs) (*1*) or diverging waves (*2*) to insonify a large field of view. These systems have previously been leveraged to create vector flow imaging (*3*, *4*), super-resolution imaging (*5*–*7*), and functional brain imaging (*8*, *9*). PW imaging, accomplished with such scanners, has the potential to improve fast, volumetric imaging. With this technology, planar waves are steered over a limited range of angles that are determined by the focal parameters. To obtain a PW resolution cell equivalent to conventional imaging, the maximum steering angle must be limited and sufficient angled PW reflections obtained to sample the object. Therefore, it is advantageous to use a large transducer aperture to minimize the angles required to interrogate a large field of view. Similarly, the presence of bone requires an extended aperture to fully surround regions.

We combine 256-channel building blocks on a research US scanner to achieve a large channel and transducer element count. This technology has the potential to be impactful across imaging with US; however, tools for fast imaging of large planes or volumes are missing. Here, we realize the utility of this platform by creating and disseminating the required BF (BF) software, integrating multiple graphics processing units (GPUs) to process the very large datasets, and developing transducer geometries and tools to explore this realm. The result is a high–channel count system that achieves a high-volume acquisition rate. We combine extended aperture transducers with these optimized acquisition tools and demonstrate enhanced image quality and speed of acquisition.

Our immediate goal is to develop the methodology to improve panoramic US imaging in applications that are typically problematic for US due to the presence of bone and tissue motion. To this end, we surround the region with eight linear transducers in an octagonal geometry, effectively rotating the US point spread function (PSF) to limit the effects of diffraction and attenuation. Imaging with the octagonal array is an extension of US tomography that typically uses circular (*10*) or conformal transducer arrays (*11*) to acquire data. In transmission tomography, one set of elements transmits the sound wave, and other transducer elements facing the transmitter receive the transmitted waves. This process is then rotated around the array, often requiring mechanical translation. Computationally intensive iterative methods such as full-waveform inversion (*12*, *13*), born inversion (*14*, *15*), and inverse scattering (*16*, *17*) are then applied to construct the image and estimate parameters. Consequently, reconstruction of one image slice requires 10 s to several hours (*10*, *18*). Here, our goal is to visualize structures and their motion on a subsecond time scale and, therefore, to acquire and process large datasets at video rates.

The musculoskeletal application is particularly challenging as the speed of sound (SOS) varies within millimeters between regions of muscle (1588 m/s), tendon (1750 m/s), and cortical bone (3514 m/s) (*19*), and additional processing is required to reduce clutter. We propose a distributed beamformer (referred to as a partial BF) to acquire a large field of view within the time limits imposed by physiological motion. We term the conventional beamformer with centralized processing of the entire datasets a global beamformer. We exclude a microbeamforming (or subarray BF) strategy since these strategies cannot implement flexible ultrafast PW imaging. The addition of (i) a modified form of coherence factor (CF) weighting during receive BF and (ii) a dual SOS correction technique during image reconstruction reduces reverberation clutter and sidelobes, respectively. The CF (*20*, *21*) weighting is modified from that implemented by previous researchers through application on each rotating view. In summary, we exploit fast acquisition of a large panoramic field of view to achieve nearly isotropic in-plane resolution and reduce the clutter floor and motion artifacts, thus improving image contrast in studies of phantoms, small animals, and human volunteers.

## RESULTS

### High–channel count and GPU-enabled partial BF facilitate practical video rate strategies

**Fig. 1.**
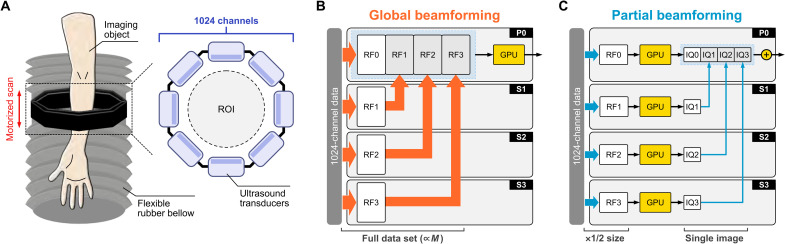
Fast volumetric US imaging system. (**A**) Schematic of the system. The imaging probe consists of eight linear transducers in octagonal geometry with 1024 elements and a circumcircle diameter of 162 mm. A 3D printed structure supports the octagon assembly that is attached to a motorized scanner and two flexible rubber bellows from the top and the bottom to translate the 1024-element aperture along the elevation direction in water, having the field of view of 130 mm in diameter by 250 mm in height. A programmable 1024-channel US platform drives the customized imaging probe. Block diagrams of (**B**) global beamformer and (**C**) partial beamformer. In the global beamformer, the full RF dataset is transferred from the data acquisition board to each node and then transferred from the secondary nodes to the primary node. In the partial beamformer, the RF data transferred to each node are half sized by decentralization, and the secondary nodes transfer only a single image to the primary node. ROI, region of interest; *M*, number of PWs.

We first evaluated the performance of the global BF and the partial BF on a customized octagonal probe consisting of eight linear arrays where each array consists of 128 elements with a central frequency of 5 MHz and a pitch of 298 μm. The octagonal imaging probe was attached to a motorized scanner and two flexible rubber bellows from the top and the bottom, so that a large (up to 130 mm in diameter by 250 mm in height) imaging object housed in US coupling medium (water) is rapidly scanned with a 1024-element aperture (Fig. 1A). PWs were consecutively transmitted by each of the eight arrays to form a 2D image, i.e., one B-mode image was generated by combining the eight rotating views from each edge of the octagon (fig. S1). The image reconstructed by delay-and-sum (DAS) method is calculated as

$$r(x,z)=\sum_{l=1}^{L}\sum_{m=1}^{M}\sum_{n=1}^{N}s_{l,m}(x,z;n)$$ (1)

where *x* and *z* are the spatial location of the image pixel; *l* and *L* (= 8) are the index and the total number of views; *m* and *M* are the index and the total number of PWs (steering angles) transmitted by each view; *n* and *N* (= 1024) are the index and the total number of the Rx channels; and *s*_*l*,*m*_(*x*, *z*; *n*) is the demodulated and delayed radio frequency (RF) signal from the *n*th Rx channel at the *m*th Tx angle and *l*th view. On the basis of an initial evaluation, we set the active number of Rx channels per each view to be 384, i.e., the channel data acquired from the transmitting array and its two neighboring arrays were used for the reconstruction, corresponding to coherence limitations of the received data (fig. S1A). Equation 1 is then expressed as

$$r(x,z)=\sum_{l=1}^{L}\sum_{m=1}^{M}\;\sum_{n\in N_{l}}s_{l,m}(x,z;n)$$ (2)

where $N_{l}={⋃}_{i=l-1}^{l+1}E_{i}$ is the set of Rx channel indices active on at the *l*th view and *E_i_* is the set of 128 local channels corresponding to the *i*th sublinear array. Note that the index *i* circularly shifts over eight arrays, i.e., *E*_0_ = *E*_8_ and *E*_9_ = *E*_1_.

The global BF has a centralized processing scheme, and, thus, the RF data are transferred from the data acquisition board to each 256-channel building block (delineated as P0 for the primary node and S1 to S3 for the secondary nodes) and then saved and processed on the primary node. Here, we define the first data transfer from the acquisition board to the four nodes as HWtoH transfer and the transfer from secondary nodes to the primary node as remote direct memory access (RDMA) transfer. The data sizes for HWtoH (*D*_1_) and RDMA (*D*_2_) in the global BF are calculated as *D*_1,global_ ∝ *N*_S_ × *M* × *L* × *N*_subCh_ and *D*_2,global_ ∝ *N_S_* × *M* × *L* × *N*_subCh_, respectively, where *N*_S_ is the number of time samples and *N*_subCh_ (= 256) is the total number of channels per node (Fig. 1B).

Our goal is to create an environment that facilitates processing of very large US datasets for video rate display by accelerating both data transfer time and data processing time. To accomplish this, we decomposed the BF process in Eq. 1 into each node as

$$\begin{array}{c} r(x,z)=\sum_{l=1}^{L}\sum_{m=1}^{M}\sum_{k=0}^{3}\;\sum_{n\in N^{k}}s_{l,m}(x,z;n)\\ =\sum_{k=0}^{3}\left\{\sum_{l=1}^{L}\sum_{m=1}^{M}\;\sum_{n\in N^{k}}s_{l,m}(x,z;n)\right\} \end{array}$$ (3)

where *k* indexes the nodes (*k* = 0,1,2,3 for nodes P0, S1, S2, and S3); and *N^k^* = {1,2, …, *N*_subCh_} + *kN*_subCh_ is the set of Rx channel indices at node *k*. Likewise, considering the active number of Rx channels per view, the computation in Eq. 3 is further reduced as

$$\begin{array}{c} r(x,z)=\sum_{k=0}^{3}\left\{\sum_{l=1}^{L}\sum_{m=1}^{M}\;\sum_{n\in N_{l}^{k}}s_{l,m}(x,z;n)\right\}\\ =\sum_{k=0}^{3}\left\{\sum_{l\in L^{k}}\;\sum_{m=1}^{M}\;\sum_{n\in N_{l}^{k}}s_{l,m}(x,z;n)\right\}\\ =\sum_{k=0}^{3}{IQ}_{k}(x,z) \end{array}$$ (4)

where *L^k^* is the set of Tx view indices actively on at node *k* and $N_{l}^{k}$ is the set of Rx channel indices actively on at node *k* and the *l*th view, i.e., $N_{l}^{k}={⋃}_{i=l-1}^{l+1}E_{i}$ if ∃*i* ∈ {2*k* + 1,2*k* + 2}, and $N_{l}^{k}=\emptyset$ otherwise. At each node, *L^k^* has a length of *L*/2 since $N_{l}^{k}=\emptyset$ for *l* ∉ {2*k*,2*k* + 1,2*k* + 2,2*k* + 3}. Last, we define *IQ_k_*(*x*, *z*) as the partially beamformed in-phase and quadrature (IQ) data at node *k*.

The data transfer sizes *D*_1_ and *D*_2_ in the partial BF are calculated as *D*_1,partial_ ∝ *N*_S_ × *M* × *L*/2 × *N*_Ch_node__ and *D*_2,partial_ ∝ *N_x_* × *N_z_*, where *N_x_* and *N_z_* are the number of reconstruction pixels in the image grid. By distributing the BF process into the local node partial reconstruction, we achieved an effective data reduction of 1/2 in *D*_1_ and 1/*FML* in *D*_2_, where *F* is the downsampling factor relating the IQ data to the RF (Fig. 1C). With partial BF, *D*_2_ is no longer dependent on the total number of PWs that range from 24 to 280 in our implementation. In a typical image acquisition, *N*_S_ = 3712, *N*_subCh_ = 256, *N_x_* = *N_z_* = 350 [one wavelength (λ) grid], and the downsampling factor *F* was 7.8 for a 1λ grid and 1.9 for a 0.5λ grid. The primary node had a low additional computational load in summing the IQ data over the four nodes, which was negligible compared to the whole process.

**Fig. 2.**
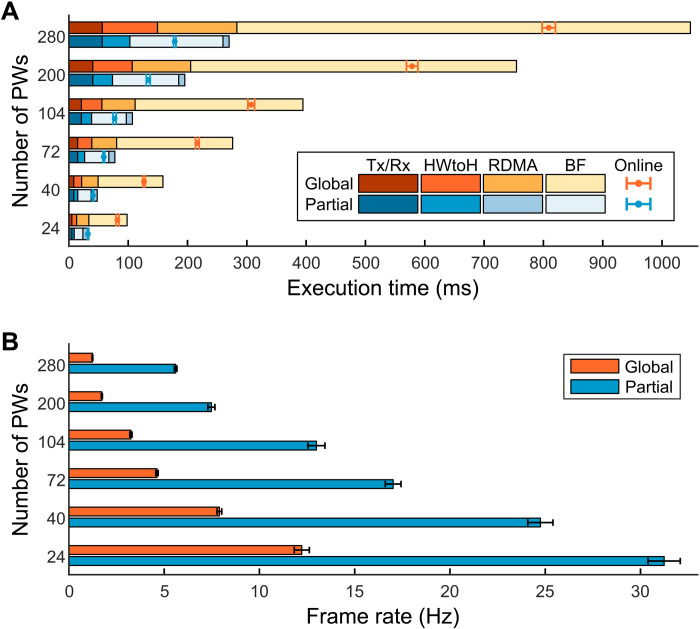
Comparison between the global and partial beamformers. (**A**) Execution time for the global and partial beamformers by process to acquire a one-frame tomographic image with a varied number of PWs. Total execution time was estimated by four consecutive processes: (i) Tx/Rx events, (ii) data transfer from the data acquisition board to the host computer (HWtoH), (iii) RDMA data transfer from a secondary system to the primary system, and (iv) image reconstruction. Other processes including synchronizing the four nodes, combining IQ data, and display do not unduly affect the frame rate. The field of view was 100 mm (*X*) × 100 mm (*Z*) with a pixel size of 1λ. The RF data size for each system was 3712 (samples) × 256 (channels) × (effective number of PWs) × 2 bytes, and the beamformed IQ data size was 350 (*X*) × 350 (*Z*) × 8 bytes (complex single precision). The pulse repetition frequency (PRF) was set to 5 kHz. The elapsed time for Tx/Rx events were determined by the PRF of insonation, which is the same for both global and partial beamformers. (**B**) Frame rate of the global and partial beamformers with a varied number of PWs.

We next evaluated the performance of the global BF and partial BF in terms of frame rate. Figure 2 shows the performance of the global BF and partial BF. The RF data size and corresponding HWtoH transfer time (∝1/*D*_1_) were reduced twofold (Fig. 2A and tables S1 and S2). Further, the RDMA transfer time (∝1/*D*_2_) in the global BF was directly proportional to the number of PWs, e.g., ~20 ms with 24 PWs and ~134 ms with 280 PWs (table S1). In the partial BF, RDMA transfer was accomplished in ~10 ms regardless of the number of PWs used (table S2). Last, the image reconstruction process was reduced fivefold using the partial BF compared to the global BF. The online frame rate was 31.2 Hz with 24 PWs and 5.6 Hz with 280 PWs, which were ~2.6× and ~4.5× improvements compared to the global BF process (Fig. 2B and tables S1 and S2). The online frame rate was faster than the sum of execution times for each process, as data acquisition was begun while RDMA transfer and reconstruction occurred (Fig. 2A and tables S1 and S2).

With (i) a reduced amount of data transferred from the secondary nodes to the primary node and (ii) distributed node-local process that leverages computing power, the partial BF approach achieved video rate display for this high–channel count system. The computing process was further accelerated through GPU implementation (*22*). Note that the partial BF process generates identical results to the global BF process for linear beamformers where the superposition principle holds (fig. S2).

### Combining partial reconstruction with nonlinear weighting to improve visualization of small structures

We then implemented nonlinear coherence weighting across the transducer aperture to reject reverberation clutter associated with strong reflectors. The image reconstructed by a DAS algorithm with CF weighting (*20*, *21*) is expressed as

$$\begin{array}{c} r_{\mathrm{DAS}+\mathrm{CF}}(x,z)=\sum_{l=1}^{L}\sum_{m=1}^{M}\left\{\left(\sum_{n=1}^{N}s_{l,m}(x,z;n)\right)\times {CF}_{l,m}(x,z)\right\}\\ =\sum_{l=1}^{L}\sum_{m=1}^{M}\left\{\left(\sum_{n=1}^{N}s_{l,m}(x,z;n)\right)\times \frac{{\left|\sum_{n=1}^{N}s_{l,m}(x,z;n)\right|}^{2}}{N\sum_{n=1}^{N}{|s_{l,m}(x,z;n)|}^{2}}\right\} \end{array}$$ (5)

In the CF calculation, we considered the received channel data from the transmitting array, i.e.

$$\begin{array}{rl} r_{\mathrm{DAS}+\mathrm{CF}}(x,z) & =\sum_{l=1}^{L}\sum_{m=1}^{M}\{\left(\sum_{k=0}^{3}\;\sum_{n\in N_{l}^{k}}s_{l,m}(x,z;n)\right)\\ & \;\times \frac{{\left|\sum_{n\in E_{l}}s_{l,m}(x,z;n)\right|}^{2}}{|E_{l}|\sum_{n\in E_{l}}{|s_{l,m}(x,z;n)|}^{2}}\} \end{array}$$ (6)

Multiplication of the CF weighting is applied before summation over PWs and views, and the resulting RDMA transfer size is *D*_2,DAS+CF,partial_ = *D*_2,DAS,partial_ + *D*_2,CF,partial_ ∝ *N_x_* × *N_z_* × *M* × (*L*/2 + 1). To speed acquisition, we defined a modified CF as the average ratio between the energy of coherent and incoherent sum in signals across the receive aperture at each view, and, thus, it suppresses incoherent signals. The averaged CF weight over PWs is

$$\begin{array}{c} r_{\mathrm{DAS}+\bar{\mathrm{CF}}}(x,z)=\sum_{l=1}^{L}\left\{\left(\sum_{m=1}^{M}\;\sum_{n\in N_{l}}s_{l,m}(x,z;n)\right)\times \left(\sum_{m=1}^{M}{\mathrm{CF}}_{l,m}(x,z)\right)\right\}\\ =\sum_{l=1}^{L}\left\{\left(\sum_{k=0}^{3}\;\sum_{m=1}^{M}\;\sum_{n\in N_{l}^{k}}s_{l,m}(x,z;n)\right)\times \bar{{\mathrm{CF}}_{l}}(x,z)\right\} \end{array}$$ (7)

The data transfer size *D*_2,partial_ is $D_{2,\mathrm{DAS}+\bar{\mathrm{CF}},\mathrm{partial}}\propto N_{x}\times N_{z}\times (L/2+1)$, and we achieved an effective data reduction of (*L*/2 + 1)/*FML* compared to the global BF. As in the DAS reconstruction, the RDMA transfer time was constant regardless of the number of PWs (slightly increased from 10.23 to 11.14 ms).

### Image quality is enhanced by multiarray acquisition and processing

**Fig. 3.**
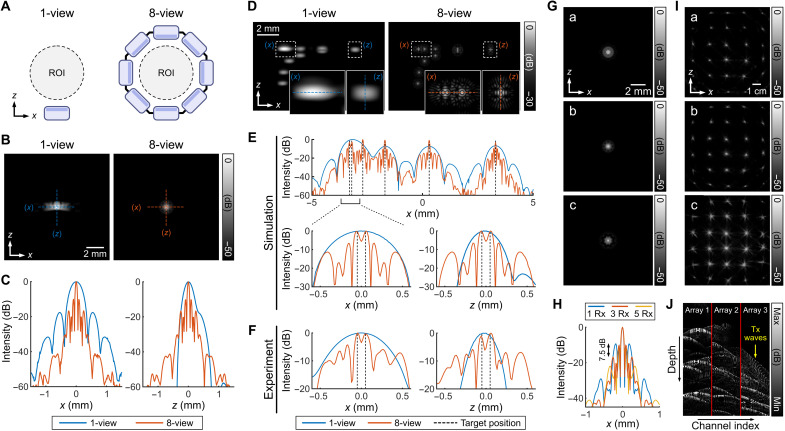
Simulations and experiments on resolution for 1-view versus 8-view. (**A**) Acquisition schemes of 1-view and 8-view. (**B**) B-mode images and (**C**) line profiles in *x* and *z* directions of the PSF for 1-view versus 8-view. A point target was located at the center of the arrays. For the 1-view acquisition, 40 PWs (−5° to 5°) were used. For the 8-view acquisition, five PWs (−5° to 5°) were transmitted for one view and the active arrays were rotated (40 PWs in total). (**D**) Simulated B-mode images of resolution targets for 1-view versus 8-view acquisitions. (**E**) Simulated and (**F**) experimental line profiles of two targets with 0.1-mm separation along the *x* and *z* axes. For the 1-view acquisition, 40 PWs (−5° to 5°) were used. For the 8-view acquisition, five PWs (−5° to 5°) were transmitted for one view and the active arrays were rotated (40 PWs in total). (**G**) Simulated B-mode images for 8-view acquisition with (a) 1 Rx, (b) 3 Rx, and (c) 5 Rx for each view. (**H**) The PSFs along the *x* direction for the varied number of receive channels. (**I**) Experimental B-mode images of the nylon wire targets for 8-view acquisition with (a) 1 Rx, (b) 3 Rx, and (c) 5 Rx for each view. (**J**) RF data of the nylon wire targets recorded by arrays 1 to 3 with array 1 as the transmitting array.

The image quality of the octagonal array (hereafter referred to as 8-view) was evaluated in terms of resolution and contrast compared to the conventional linear array (hereafter referred to as 1-view) (Fig. 3A). We first evaluated the PSF based on simulation. For the 8-view acquisition, the full width at half maximum (FWHM) spatial resolution was isotropic in the center of the array at 0.05 mm, compared with 0.32 and 0.23 mm for *x* and *z* resolutions, respectively, for the 1-view acquisition (Fig. 3, B and C, and table S3).

We further compared the ability to resolve nearby targets for 1-view versus 8-view acquisitions (Fig. 3D). We found upon simulation (Fig. 3E) and experiment (Fig. 3F) that targets separated by 100 μm in the *x* or *z* direction could be resolved using the 8-view acquisition but not in the *x* or *z* direction using the 1-view acquisition. Simulation results also showed that the expanded aperture afforded by multiple arrays on receive suppressed grating lobes (Fig. 3, G and H). We compared the scenarios of 1 Rx (*N_l_* = ⋃_*i* = *l*_*E_i_*), 3 Rx ($N_{l}={⋃}_{i=l-1}^{l+1}E_{i}$), and 5 Rx ($N_{l}={⋃}_{i=l-2}^{l+2}E_{i}$) arrays at each view. Grating lobes were apparent with a height of −9.3, −16.8, and −21.9 dB for 1 Rx, 3 Rx, and 5 Rx, respectively (Fig. 3G and table S3). The experimental results showed that the expanded aperture from 1 Rx (Fig. 3I, a) to 3 Rx (Fig. 3I, b) suppressed grating lobes; with 5 Rx (Fig. 3I, c), however, the arrays at the two extremities received transmitted waves from the Tx array directly (Fig. 3J). Therefore, 3 Rx and 8-views were applied for our acquisitions.

**Fig. 4.**
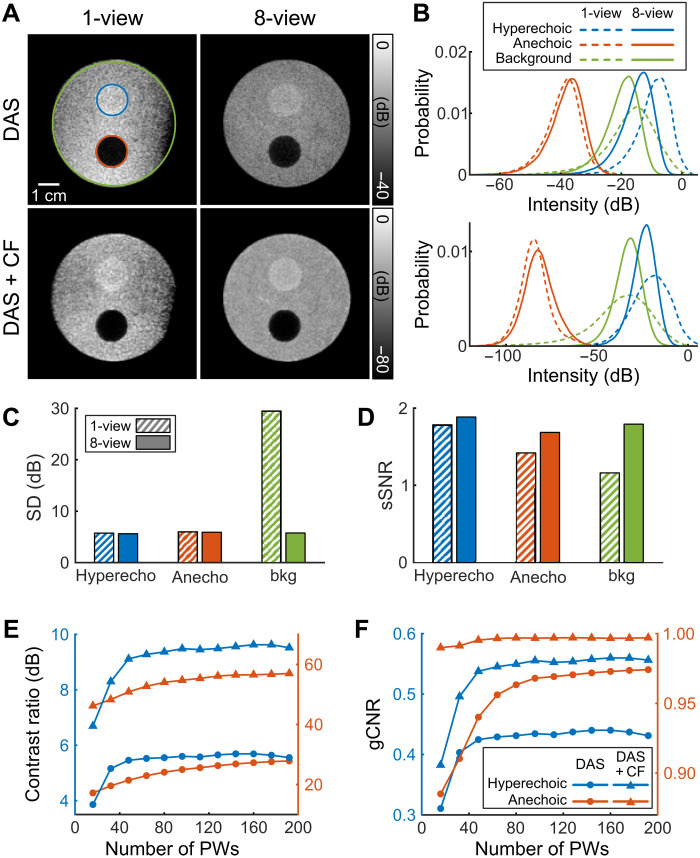
Quantitative analysis on a contrast phantom. (**A**) B-mode images of a phantom containing hyperechoic and anechoic inclusions obtained from 1-view (24 PWs between −12.5° and 12.5°) and 8-view (3 PWs between −12.5° and 12.5° per view, 24 PWs in total) acquisitions reconstructed by DAS and DAS with averaged CF with a pixel size of 0.1λ. The hyperechoic and anechoic regions are marked by blue and red circles, respectively, and the background region corresponds to the green circle region excluding the hyperechoic and anechoic regions in (A). In the case of the CF-weighted images, the dynamic range was doubled to account for an implicit squaring effect. An array directed upward from the bottom of the images was used for 1-view acquisition. (**B**) Comparison of the probability density function of the intensity values in the hyperechoic, anechoic, and background regions for DAS-based images (top) and DAS-with-CF-based images (bottom). Quantification on the hyperechoic and anechoic cysts in (A): (**C**) SD, (**D**) sSNR, (**E**) CR, and (**F**) gCNR. bkg, background.

Coherent imaging with the full aperture also improved image quality. With the 1-view geometry, the full phantom could not be imaged, the mean background intensity varied, and the speckle texture was coarse (Fig. 4A). As a result, the SD of the intensity was greater with 1-view acquisition than with 8-view for each region of the image (Fig. 4C and table S4). The 8-view acquisition also improved the speckle signal-to-noise ratio (sSNR) (*23*) compared to the 1-view acquisition. An increase in sSNR from 1.78 to 1.89 was observed for the hyperechoic region and 54% (from 1.16 to 1.79) in the background, comparing the 8-view and 1-view acquisitions (Fig. 4D and table S4).

We then compared the images reconstructed by DAS, DAS with the original CF in Eq. 5, and DAS with the averaged CF in Eq. 7 (fig. S3A). In the results, the CF weighting refers to the averaged CF unless otherwise specified. The contrast ratio (CR) was improved by adding CF weighting and further improved with the averaged CF in both hyperechoic and anechoic regions (fig. S3B and table S5). The generalized contrast-to-noise ratio (gCNR) showed the same trend in the hyperechoic region. The gCNRs in anechoic region with the original and averaged CF were similar, achieving 1.00 when more than 40 PWs were used (fig. S3C and table S5). By modifying CF weighting, we accelerated the processing speed via partial reconstruction and improved the image quality. The combined use of CF and 8-view acquisition improved visualization of the hypoechoic cyst, reducing apparent noise intensity within the region. The 8-view acquisition reduced the SD of the intensity in the hyperechoic, anechoic, and background regions. The improvements obtained with 8-view acquisition are illustrated by the comparison of the probability density function of intensity values (Fig. 4B).

Last, we evaluated the impact of the number of PWs used for acquisition of the phantom images as a function of the CR (Fig. 4E) and gCNR (Fig. 4F). The greatest improvement in the CR and gCNR for the hyperechoic target occurred as the number of PWs increased from 16 to 32. For the anechoic target, a gradual increase in CR and gCNR was observed with increasing numbers of PWs.

### Small animal imaging validated the improved spatial resolution and field of view

**Fig. 5.**
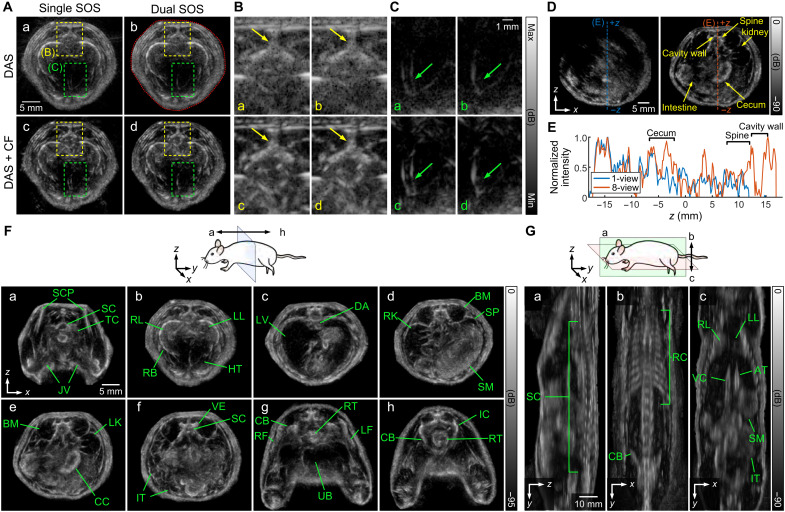
US tomography of rodent anatomy. (**A**) Experimental comparison of cross-sectional images of the rat thorax obtained with the octagonal array and reconstructed using a single SOS value and using dual SOS values reconstructed by DAS and DAS with the averaged CF was achieved by correcting the delays for the pixels falling inside the segmented area (red dotted line in b) from the image reconstructed using a single SOS value. Dynamic range is set to cover the highest intensity 90% of pixels. (**B**) Zoom over the location of the vertebrae (yellow arrows) outlined in (A) in yellow. (**C**) Zoom over the location of the outer heart wall (green arrows) outlined in (A) in green. (**D**) Experimental comparison of the cross-sectional image of a rat abdomen imaged with the octagonal array with 1-view acquisition (left) and 8-view acquisition (right). For 1-view, the array was placed vertically at the bottom of the image. (**E**) The line profiles for 1-view versus 8-view extracted from images in (D). (**F**) Cross-sectional images of (a) upper and (b) lower thoracic cavity, (c) two lobes of the liver, (d) upper and (e) lower abdominal cavity, (f) abdominopelvic cavity, and (g) upper and (h) lower pelvic cavity in the rat. (**G**) Cross-sectional images of the (a) sagittal and (b and c) coronal view of the rat trunk. AT, aorta; BM, backbone muscles; CB, coxal bone; CC, cecum; DA, dorsal aorta; HT, heart; IC, ischium; IT, intestines; LF, left femur; LK, left kidney; LL, left lung; LV, liver; JV, jugular vein; RB, rib; RC, rib cage; RF, right femur; RK, right kidney; RL, right lung; RT, rectum; SC, spine; SCP, scapula; SM, stomach; SP, spleen; TC, trachea; UB, urinary bladder; VC, vena cava; VE, vertebra.

We evaluated the impact of the enhanced resolution and extended field of view in imaging of a rat model (Fig. 5). While typical systems for small animal imaging use a high center frequency (typically greater than 40 MHz) to achieve high-resolution imaging, the transducer and system developed here can use a clinically relevant US frequency to achieve submillimeter resolution and distinguish anatomical features throughout the animal. A particular challenge with imaging through a water bath was the impact of water temperature on phase aberration and strong reflections on the surface of the imaging target. To alleviate the artifacts, we added correction for the SOS in the surrounding water, with the SOS map applied (red dotted line in Fig. 5A, b). This was performed with a beamformer that incorporated the SOS of water surrounding the sample (1465 m/s) and the SOS for the sample (1510 m/s). Accounting for the differences enhanced the visualization of small structures such as the vertebrae (Fig. 5A, a and b). Applying CF weighting increased the contrast between the highly coherent bone and the surrounding tissue (Fig. 5A, c and d). To facilitate the comparison between images with and without CF, we used a different dynamic range for the images without CF to ensure an approximately equivalent histogram of pixel grayscale levels in the images. Although many anatomical structures are distinguishable in the images reconstructed with a single SOS, fine anatomical details were not easily visualized because of the delay estimation error inside the body. For instance, the vertebrae were enlarged, the location of the dorsal aorta was not obvious, and the heart wall boundaries were doubled (Fig. 5, B and C). Application of the dual SOS beamformer resolved these artifacts.

With the 8-view acquisition, the cross section of the rat abdomen was imaged and reconstructed with high fidelity. As a result, the spine and the abdominal cavity, including the left kidney, cecum and small intestines, were clearly depicted (Fig. 5D). With 1-view acquisition, the reconstruction was incomplete, and the images lack fidelity due to poor image quality and the limited field of view (Fig. 5D). Direct comparison of the profiles (Fig. 5E) in the sagittal plane shows the improved discrimination of anatomical features with the multiarray reconstruction.

Using 8-views, dual SOS beamformer, and CF weighting, high-fidelity images of the anatomical structures in the cervical and thoracic (Fig. 5F, a and b), abdominal (Fig. 5F, c to f), and pelvic (Fig. 5F, g and h) cavity of the rat were successfully reconstructed. The trunk diameter was ~4.4 cm (Fig. 5F, g and h). Anatomical features, including trachea, ribs, vertebrae, scapula, heart, liver, lungs, major vessels (jugular vein and dorsal aorta), kidneys, bladder, femur, rectum, etc., were easily identified (Fig. 5F). Sagittal and coronal slices of the 3D reconstructed volume delineated the entire spinal cord (Fig. 5G, a and b), rib cage (Fig. 5G, b), and anatomical location of the lungs, vena cava, aorta, stomach, and intestines (Fig. 5G, c).

### Extended aperture methodology applied to human musculoskeletal imaging

**Fig. 6.**
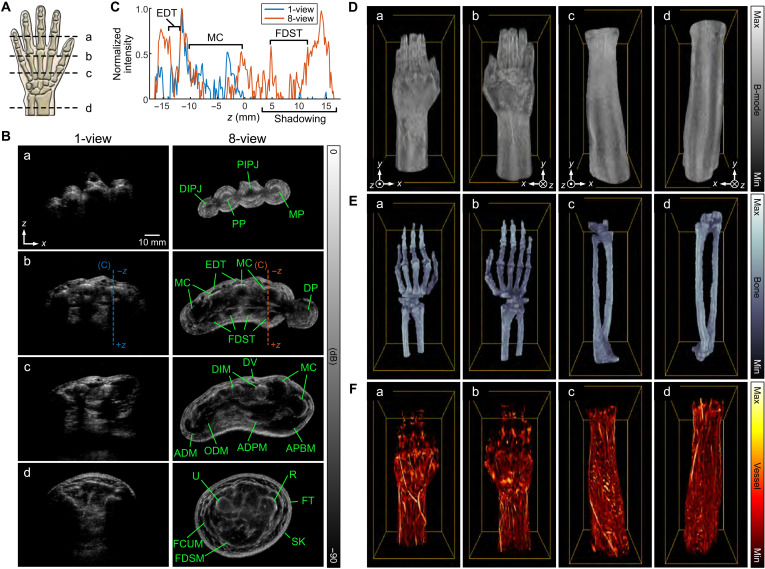
In vivo images of the human hand, wrist, and forearm. (**A**) Schematic for the position of acquisition. (**B**) Cross-sectional images with 1-view (left column) versus 8-view (right column) for (a) distal hand (finger) section; (b) distal metacarpal section; (c) proximal metacarpal section; and (d) distal forearm section. All images were reconstructed using the dual SOS beamformer with CF weighting at a pixel size of 0.5λ. (**C**) The line profiles for 1-view versus 8-view extracted from images in (B, b). (**D** to **F**) Volume rendered images of the human (a and b) hand-wrist and (c and d) forearm region. The yellow bounding boxes represent the acquired volume of 120 mm (*X*) × 80 mm (*Z*) × 250 mm (*Y*) and 100 mm (*X*) × 100 mm (*Z*) × 250 mm (*Y*), respectively, which acquired in 12.5 s. (D) B-mode, (E) bone, and (F) vessel images are presented in gray, bone, and hot colormap, respectively. ADM, abductor digiti minimi muscle; ADPM, adductor pollicis muscle; APBM, abductor pollicis brevis muscle; DIM, dorsal interosseous muscle; DIPJ, distal interphalangeal joint; DP, distal phalanx; DV, dorsal vein; EDT, extensor digitorum tendon; FCUM, flexor carpi ulnaris muscle; FDSM, flexor digitorum superficialis muscle; FDST, flexor digitorum superficialis tendon; FT, fat; MC, metacarpal; MP, middle phalanx; ODM, opponens digiti minimi muscle; PIPJ, proximal interphalangeal joint; PP, proximal phalanx; R, radius; SK, skin; U, ulna.

We then applied this technology in imaging of the human hand, wrist, and forearm (Fig. 6A), and the results were consistent with the comparisons for phantom and rodent imaging. Applying CF weighting increased the contrast of anechoic regions (fig. S4A). Contrast increased across line profiles of the dorsal vein by 36.4 and 41.9 dB with the original CF and averaged CF weightings, respectively, as compared with standard DAS imaging (fig. S4B). The greatest improvement in contrast for an extensor digitorum tendon (44.5 dB) was achieved by applying averaged CF weighting, compared with a 38.9-dB enhancement with the original CF weighting (fig. S4C).

With 1-view acquisition, less than 50% of the cross-sectional area was visualized, and the images lack fidelity due to poor image quality, limited field of view, and shadowing artifacts (left column in Fig. 6B). With 8-view acquisition, the entire cross section was reconstructed, the boundaries of the radius and ulna were well defined, and tendons and superficial vasculature and veins were detected (right column in Fig. 6B). The comparison of line profiles clearly shows the enhanced discrimination of anatomical features using 8-views (Fig. 6C). Cross-sectional images of the human distal hand (fingers) (Fig. 6B, a), mid hand (Fig. 6B, b and c), and distal forearm (Fig. 6B, d) were acquired. The largest dimension in these images was ~9.4 cm in the mid hand section (Fig. 6, b) where the entire field of view cannot be imaged with conventional arrays. The 8-view imaging was robust to acoustic shadowing effects from the bone (movies S1 and S2). Anatomical structures including bones, interphalangeal joints, superficialis tendons, muscles, radius, and ulna were identified (Fig. 6B). With 40 PWs, we could clearly visualize the detailed anatomy and motion at 25 Hz (movie S3). The performance improved with additional PWs, enhancing visualization of anechoic targets, presumably due to lateral and axial grating lobe reductions (fig. S5, A and B). For example, image contrast of the dorsal vein improved as the number of PWs increased (fig. S5C).

We achieved a rapid acquisition of 10 cm × 10 cm × 25 cm volume during a single breath-hold with a goal to minimize the physiological motion artifacts in the large volume. To maximize the volume acquisition rate, the entire scan of 25 cm (0.5-mm step, 500 frames) was acquired and stored at a frame rate of 40 Hz using 40 PWs without reconstruction/display, and, thus, the volumetric data were acquired in 12.5 s. This can be further accelerated to 9.5 s using 24 PWs. The hand-wrist (Fig. 6D, a and b) and forearm (Fig. 6D, c and d) regions of a volunteer were successfully acquired without detectable physiological motion. The bony skeleton (Fig. 6E) was manually segmented using the 3D Slicer platform (*24*), and the vascular volume fraction (Fig. 6F) was automatically segmented using a Hessian-based Frangi vesselness filter (*25*). All 3D volumes were rendered using 3D PHOVIS (*26*).

## DISCUSSION

We set out to develop a high–channel count system to enhance human US imaging by extending the field of view and achieving video rate acquisition of the resulting panoramic or volumetric images. We believe that this is the first demonstration of such a fast, panoramic system to image a human limb, and we find that both enhanced resolution and acquisition speed are key elements of a practical strategy for large field-of-view US imaging. The possible applications, geometries, and processing schemes are limitless. These strategies address the need to broadly improve the intuitive utility of US and to reduce the operator dependency that is a major limitation of the technology. Through the combination of the large 5-MHz transducer aperture (47 cm in circumference), distributed BF, and nonlinear processing, the volume was acquired in a single breath-hold with sufficient fidelity to segment both the bone structure and vascular compartment of the limb. Consistent with previous work on improving image quality by increasing effective transducer aperture size (*23*, *27*–*29*), we showed improvements in image resolution with the 8-view large-aperture acquisition. Using a fully coherent imaging strategy with the octagonal array, it was feasible to achieve a narrow −6-dB PSF (50 μm), to resolve in-plane targets that were separated by 100 μm in phantom measurements, and to visualize submillimeter blood vessels and structures in rodent anatomy and throughout the human limb. The ability to achieve high-resolution imaging with a clinically relevant center frequency was therefore demonstrated to have utility in both small animal and human imaging. Visualization of small features also requires reduction in the background SD; here, the background variation was reduced by more than 20 dB with coherent processing of 8-views. Automatic segmentation of the vasculature was then achieved using the full tomographic field of view by implementing a vesselness filter to enhance tubular structures (*30*, *31*).

The high–channel count data and fast processing were exploited in nonlinear coherence weighting of the received echoes obtained as a function of the angle of incidence. Reverberation is particularly strong in imaging of the hand and wrist due to the distribution of bone and cartilage, suggesting that such processing is important for this application, and we found that such weighting enhanced the gCNR in this musculoskeletal application. The averaged coherence weighting processing was compatible with the partial BF process. Other nonlinear weighting schemes will be explored in future work, as it is feasible to design custom weighting protocols for varied tissue types. For practical human imaging with submillimeter resolution, acquisition speed is also important. Here, reasonable CR and gCNR images were obtained with 40 PWs and increasing the number of PWs further enhanced image quality. The frame rate achieved for 40 PWs was 24 Hz, and, therefore, a stack of 100 images was acquired in seconds. With the high-speed acquisition, the segmentation of both highly reflective bone and poorly echogenic blood vessels was feasible with a full dynamic range of ~90 dB and a submillimeter isotropic in-plane resolution. Small structures such as the dorsal vein and extensor digitorum tendon were detected, and the muscle could be segmented. These promising results show the potential of our system for orthopedic and myopathic imaging (*32*). To the best of our knowledge, this has not been accomplished previously and offers a substantial benefit for the evaluation of the individual tissue compartments.

In addition to increasing the aperture circumference, the system developed here incorporates 1024 receive channels, >8 gigabytes of memory and dedicated GPUs behind each 256-element subaperture. This combination facilitates acquiring and processing PWs from the entire large aperture. The time required to fully sample a large aperture with conventional focused beams or fully mechanically steered systems is prohibitive, particularly in regions of moving tissue. Here, each PW interrogated ~3.8 cm in azimuth with the central targets interrogated with each PW. The partial BF scheme was implemented on the GPU and improved the frame rate with distributed computing by reducing the computational burden and the size of transferred and stored data. Links to these tools are provided in the Data and Materials Availability statement under Acknowledgments. The implementation of partial aperture processing allowed for real-time display of volumetric data and also facilitated advanced processing.

The high–channel count, distributed-processing US technology developed here can also be applied to image the abdomen. For abdominal applications, a 2-MHz center frequency would be preferred, yielding a wavelength of 770 μm. The longer wavelength, as compared with 5 MHz, reduces the number of elements required to achieve a large aperture (e.g., 256 elements could achieve an aperture of 9.8 cm at half-lambda spacing). However, for the larger imaging depth required in the abdomen (>10 cm), elevational focusing is needed for a high-quality image, and, therefore, a 2D array with integrated electronics would be desirable. The development of these transducers and electronics is a focus of our team for this future application (*33*).

The large “panoramic” field of view addresses a common limitation of small field-of-view sonography by providing an intuitive display/overview of anatomic structures while maintaining high spatial resolution. Further, the large field-of-view images allow for analysis of volumetric features that are considered important in the assessment of healthy and disease states. With a wide field of view, 3D images of lesions are acquired with a simultaneous view of major anatomical landmarks, facilitating both communication between members of the health care team and repeated imaging of suspicious lesions over time. Last, there is an enormous spectrum of US-mediated therapeutics that are on the horizon, spanning ablative therapies (*34*), drug and gene delivery (*35*), opening of the blood brain barrier for the treatment of neurodegenerative disease (*36*), and the use of sonogenetics (*37*) to modulate cell-based therapeutics in situ. All of these technologies could benefit from video-rate volumetric views, whereas the conventional US imaging or tomography lacks either wide field of view or high volume rate. For the musculoskeletal system, volumetric measures of muscle are important in the analysis of sarcopenia (*38*) (broadly defined as decreased muscle strength, mass, and quality), large masses, or fluid collection in soft tissues. Quantitation of these scenarios and tracking the results over time require 3D acquisition. Currently, quantification of muscle mass or quality with diagnostic imaging requires patients to undergo computed tomography or magnetic resonance imaging, and, therefore, few patients are screened sufficiently early to allow effective intervention, although interventions are now on the horizon (*39*, *40*). With PW or diverging wave-based methods developed for Doppler (*41*), color flow (*3*, *4*), US attenuation (*42*) and SOS imaging (*43*–*45*), and elastography (*1*, *46*), we foresee expanding our platform to include these features.

In summary, we believe that creating US imaging methodologies with a high channel count, fast processing, and a large aperture has the potential to advance the use of US imaging and therapy in important clinical applications.

## MATERIALS AND METHODS

### System infrastructure

A programmable 1024-channel US platform was used to drive a customized transducer with 1024 elements. The four 256-channel nodes (Vantage 256, Verasonics Inc., Kirkland, WA) synchronized Tx/Rx channels by sending clock signals to the four nodes with a GPU (RTX Titan, Nvidia, Santa Clara, USA) in each node. The transducer consisted of eight 128-element linear arrays (L7-4, ATL/Philips, Amsterdam, Netherlands; 5-MHz central frequency, 298-μm pitch) hosted in 3D printed subassembly pieces forming an octagonal manifold. A custom container consisting of flexible rubber bellows (McMaster-Carr, Elmhurst, IL), aluminum extrusion (MISUMI, Schaumburg, IL), and 3D printed structure was constructed to support the ring assembly that was attached on two linear translation stages driven by a motor controller (ESP 300, Newport, Irvine, CA, USA) (Fig. 1A).

### Imaging sequence and image reconstruction

A dual SOS beamformer was implemented by reconstructing the image with a single SOS and then partitioning to two domains with two SOS values. With the assumption that US rays travel straight from the source to the detectors, the paths along which the rays travel inside the subject were calculated to correct for the delay errors caused by the SOS difference in the object.

### Data transfer and processing time

The execution time of HWtoH transfer and RDMA transfer was estimated on the basis of the maximum data transfer rate of 6.6 and 4.0 gigabytes/s, respectively, with additional operating offset. Image reconstruction process was divided into and analyzed by three steps: (i) copying memory from host [central processing unit (CPU)] to device (GPU) and vice versa, (ii) demodulation, and (iii) BF. All processes were measured 100 times and then averaged in the host PC for the primary node (CPU: Intel Xeon Gold 6136 Processor, GPU: NVIDIA TITAN RTX). The online frame rate was measured for a 10-cm × 10-cm image accounting for data transfer, processing, and display (tables S1 and S2).

### Image quality metrics

We used the following metrics to evaluate the image quality of our platform: (i) spatial resolution, defined as the FWHM of the PSF; (ii) CR = 20 log_10_(μ_0_/μ_1_), where μ_0_ and μ_1_ are the mean values of the envelope signal in regions 0 and 1, respectively; (iii) sSNR = μ_0_/σ_0_, for region 0; and (iv)$1-\int_{-\infty }^{\infty }\underset{x}{\min}\{p_{0}(x),p_{1}(x)\}dx$, where *p*(*x*) indicates the probability density function within the region.

### Resolution evaluation

A point target was imaged in experiments and simulations to evaluate the spatial resolution of the system (Fig. 3, B and C). The simulations were carried out using the Verasonics Research Ultrasound Simulator (*47*), with a point target of strong reflectivity defined at the center of the array. The distance between the point targets to the arrays was 7.5 cm. For the 8-view acquisition, five PWs were transmitted per view (−5° to 5°, 40 PWs in total) to image the point target. For comparison, the same target was also imaged with 1-view using 40 PWs (−5° to 5°) to ensure that same amount of illumination was received. For the experiments, a tungsten wire (25 μm in thickness) was vertically suspended in the water tank and imaged using the same sequences. To evaluate the ability to resolve nearby targets, resolution targets with 0.1-, 0.5-, 1.0-, 2.0-, and 4.0-mm separations in the *x* and *z* directions were defined at the center of the arrays and imaged in simulation (Fig. 3, D and E). For the experiments, a tungsten wire (25 μm in thickness) was hung on a two-axis (*x* and *z*) linear stage, vertically suspended in the water tank, positioned at the center of the arrays, and imaged (Fig. 3F). Then, the wire was imaged again after being moved in 0.1-mm steps from 0.0 mm to 1.0 mm in the *x* or *z* direction. Two sets of RF data were summed to mimic two nearby wire targets due to the lack of a calibrated commercial phantom for cross-sectional views. Nylon wire grid targets (50 μm in thickness) were also imaged with 1, 3, and 5 arrays used in reception (Fig. 3I).

### Phantom synthesis

Image quality metrics including CR and gCNR were evaluated on an agarose-based tissue-mimicking phantom with hyperechoic and anechoic cylindrical inclusions (Fig. 4A). The background substrate of the phantom was prepared by dissolving 1.5% (w/v) agarose powder (A10752, Alfa Aesar) in degassed water at 80°C and mixed with 1% (w/v) silicon carbide (SiC) powder (A16601, Alfa Aesar) homogeneously using a magnetic stirrer (SH-2, Faithful) before solidification. The hyperechoic inclusion [1.5% (w/v) agar and 2% (w/v) SiC] was prepared following the same procedure as for preparing the background substrate. The anechoic inclusion was filled with water. Comparison was made following the same settings for the point target experiments described above.

### Small animal and human imaging protocols

All animal experimental procedures were performed in accordance with protocols approved by the local Institutional Animal Care and Use Committee. A 7-week-old female rat (200 g of body weight) was anesthetized with a vaporized isoflurane (1 liter/min of oxygen and 2% isoflurane) gas system, and the hair was removed using clippers and depilatory cream. The rat was humanely euthanized and placed in the water tank for imaging. In total, 130 slices were acquired (covering 130 mm), and 56 PWs (8-view acquisition, 7 PWs per view, −11.5° to 11.5°) were used for imaging each slice.

A healthy female volunteer (31 years old) was consented for the in vivo imaging of the forearm, wrist, and hand. All imaging procedures followed the protocol approved by the Stanford Institutional Review Board (protocol #44593). Imaging was performed with 5 to 73 PWs per view (−12.5° to 12.5°). For the rapid volume acquisition of the human hand-forearm region, 500 slices were acquired to cover a 250 mm distance with 0.5 mm interval at a constant speed of 18.8 mm/s. Before human imaging, the transducer acoustic output was measured to ensure the imaging sequences remained within the U.S. Food and Drug Administration recommendations.
