# Supplementary material for: Fast volumetric ultrasound facilitates high-resolution 3D mapping of tissue compartments
Source: Sci Adv. 2023 May 31;9(22):eadg8176. doi: 10.1126/sciadv.adg8176 (PMC10413648; doi:10.1126/sciadv.adg8176)
Supplement: Supplementary file 1 — Figs. S1 to S5 Tables S1 to S5 Legends for movies S1 to S3 [file sciadv.adg8176_sm.pdf]

Science Advances
AAAS

Supplementary Materials for

Fast volumetric ultrasound facilitates high-resolution 3D mapping of tissue compartments

Eun-Yeong Park *et al.*

Corresponding author: Katherine W. Ferrara, kwferrara@stanford.edu

Sci. Adv. **9**, eadg8176 (2023)
DOI: 10.1126/sciadv.adg8176

The PDF file includes:

Figs. S1 to S5
Tables S1 to S5
Legends for movies S1 to S3

Other Supplementary Material for this manuscript includes the following:

Movies S1 to S3

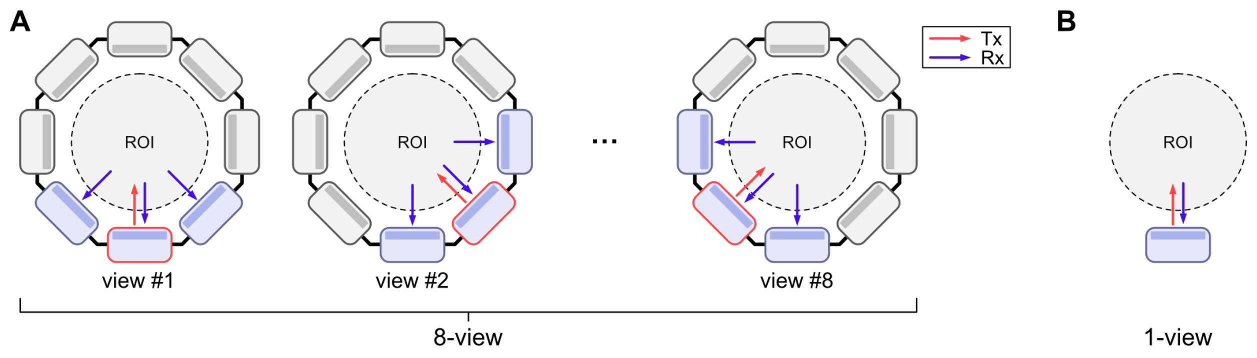

Fig. S1.

Transmit and receive configuration for B-mode imaging. (**A**) 8-view acquisition. (**B**) 1-view acquisition. Tx, transmit; Rx, receive; ROI, region of interest.

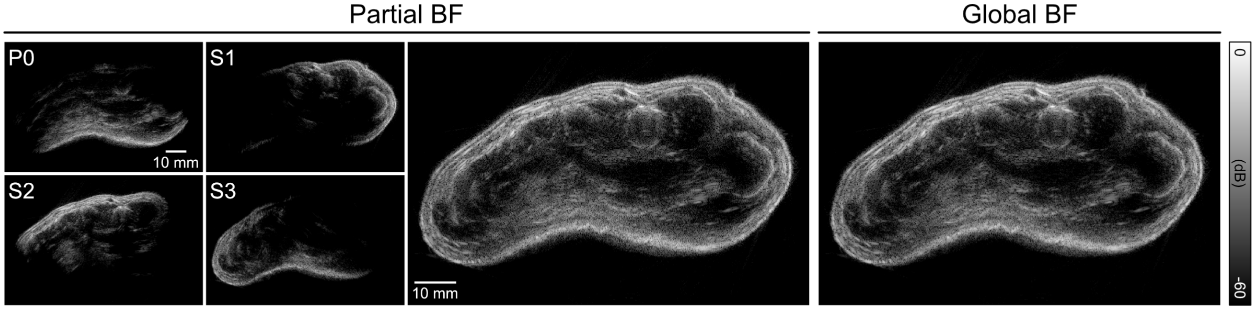

Fig. S2.

B-mode images from the partial beamformer and the global beamformer. P0-S3 represents partially reconstructed B-mode images from each node. BF, beamformer; P0, primary node; S1-3, secondary nodes.

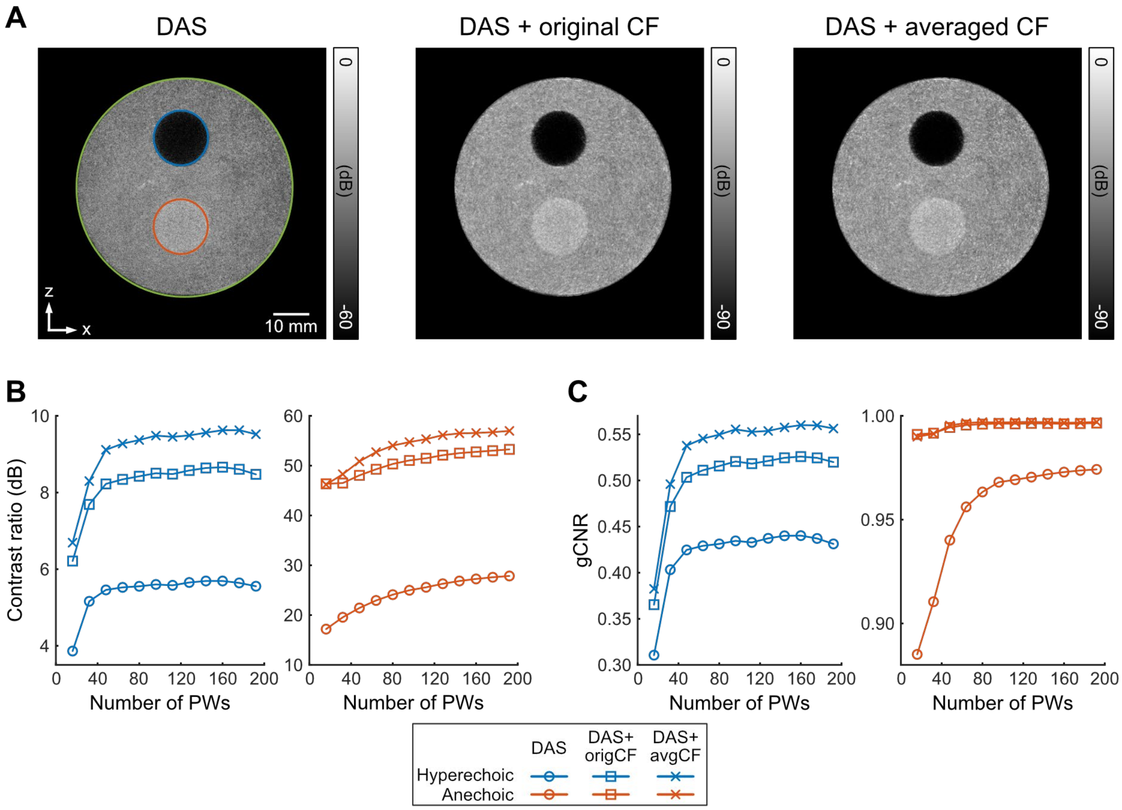

Fig. S3.

Quantitative analysis on a contrast phantom. (**A**) B-mode images of a phantom containing hyperechoic and anechoic inclusions reconstructed by DAS, DAS with original CF, and DAS with averaged CF. The hyperechoic and anechoic regions are marked by blue and red circles, respectively, and background region corresponds to the green circle region excluding the hyperechoic and anechoic regions. Quantification of contrast on the hyperechoic and anechoic cysts in (A) as a function of the number of plane waves (-12.5° to 12.5° per view). (**B**) Contrast ratio and (**C**) generalized contrast-to-noise ratio (gCNR). DAS, delay-and-sum; CF, coherence factor; PW, plane wave.

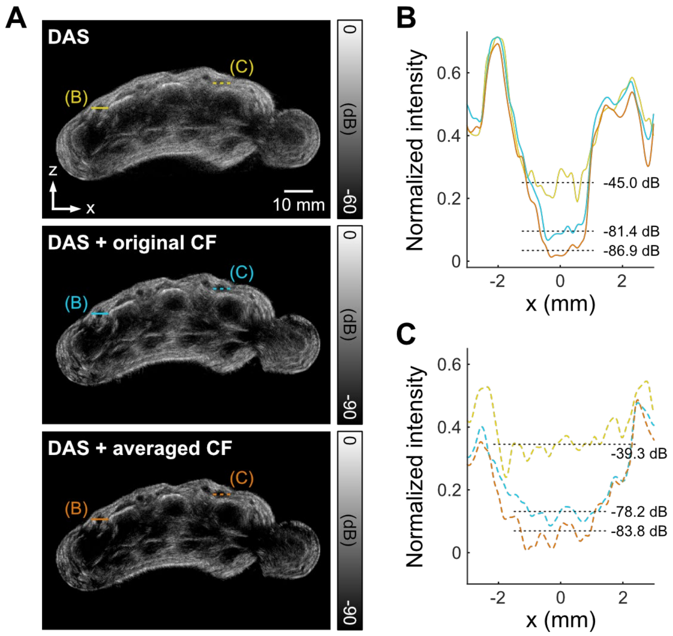

Fig. S4.

***In vivo* images with different reconstruction methods.** (**A**) Cross-sectional images for distal metacarpal section reconstructed by DAS, DAS with original CF weighting, and DAS with averaged CF weighting. (**B**) The line profiles across a dorsal vein extracted from images in (A). (**C**) The line profiles across an extensor digitorum tendon extracted from images in (A). DAS, delay-and-sum; CF, coherence factor.

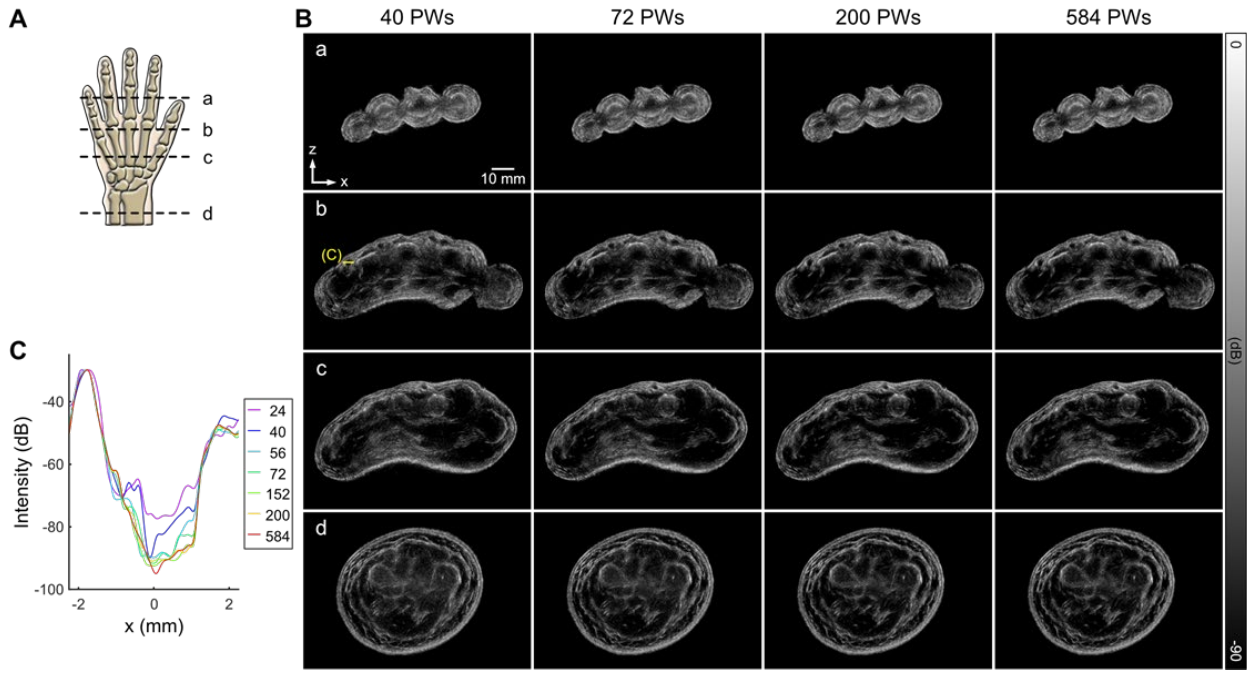

Fig. S5.

Cross-sectional images of the human hand, wrist and forearm. (**A**) Schematic for the position of acquisition. (**B**) B-mode images with various number of plane waves. Columns from left to right: 40, 72, 200, and 584 PWs between -12.5° and 12.5°, respectively. Rows include different locations: (a) distal hand (finger) section; (b) distal metacarpal section; (c) proximal metacarpal section; (d) distal forearm section. All images were reconstructed with CF and a pixel size of 0.5 wavelength. (**C**) The line profiles extracted from a dorsal vein (yellow line in B-b) with various number of plane waves. PWs, plane waves. CF, coherence factor.

Table S1.

Execution time of global beamformer. Execution time of global beamformer for the tomographic array by process with various number of plane waves (PWs). Each process was measured 100 times. Abbreviations: PW, plane wave; Tx/Rcv, transmit-receive; HWtoH, data transfer from Vantage hardware (HW) to host computer; RDMA, remote direct memory access; HtoD, memory copy from host (CPU) to device (GPU); Demod, demodulation; BF, beamformation; DtoH, memory copy from device (GPU) to host (CPU).

No. of PWs	Tx/Rcv	HWtoH	RDMA	Reconstruction				Total	Online
				HtoD	Demod	BF	DtoH		
24	5.00 ms	7.98 ms	20.62 ms	36.84 ms	7.80 ms	19.53 ms	0.31 ms	98.08 ms	81.90 ms (12.22±0.39 Hz)
40	8.20 ms	13.30 ms	27.70 ms	62.85 ms	13.03 ms	33.24 ms	0.27 ms	158.59 ms	126.60 ms (7.90±0.12 Hz)
104	21.00 ms	34.59 ms	56.02 ms	161.25 ms	33.68 ms	87.80 ms	0.27 ms	394.61 ms	307.39 ms (3.25±0.06 Hz)
280	56.20 ms	93.13 ms	133.90 ms	430.19 ms	89.73 ms	244.26 ms	0.29 ms	1047.71 ms	809.00 ms (1.24±0.02 Hz)
584	N/A								

Table S2.

Execution time of partial beamformer. Execution time of the partial beamformer for the tomographic array with a varied number of plane waves (PWs). Each process was measured 100 times. Abbreviations: Tx/Rcv, transmit-receive; HWtoH, data transfer from Vantage hardware (HW) to host computer; RDMA, remote direct memory access; HtoD, memory copy from host (CPU) to device (GPU); Demod, demodulation; BF, beamformation; DtoH, memory copy from device (GPU) to host (CPU).

No. of PWs	Tx/Rcv	HWtoH	RDMA	Reconstruction				Total	Online
				HtoD	Demod	BF	DtoH		
24	5.00 ms	3.99 ms	10.23 ms	4.57 ms	2.66 ms	6.51 ms	0.79 ms	33.74 ms	32.03 ms (31.24±0.84 Hz)
40	8.20 ms	6.65 ms	10.23 ms	7.63 ms	4.26 ms	9.94 ms	0.82 ms	47.72 ms	40.44 ms (24.75±0.65 Hz)
104	21.00 ms	17.30 ms	10.23 ms	20.29 ms	11.24 ms	26.02 ms	0.96 ms	107.54 ms	77.07 ms (12.99±0.44 Hz)
280	56.20 ms	46.57 ms	10.23 ms	54.25 ms	30.04 ms	71.75 ms	0.99 ms	270.04 ms	178.40 ms (5.61±0.07 Hz)
584	117.00 ms	97.13 ms	10.23 ms	111.51 ms	62.48 ms	152.27 ms	1.03 ms	551.64 ms	354.14 ms (2.82±0.03 Hz)

Table S3.

Resolution analysis on a point target. Evaluation on full-width at half-maximum (FWHM) of a point target for 1-view versus 8-view acquisitions and grating lobe level of a point target with the various sizes of receive aperture.

FWHM		Grating lobe		
1-view	8-view	1Rx	3Rx	5Rx
(x) 0.32 mm<br>(z) 0.23 mm	(x) 0.05 mm<br>(z) 0.05 mm	-9.3 dB	-16.8 dB	-21.9 dB

Table S4.

Quantitative analysis on a contrast phantom with 1-view versus 8-view acquisitions. Summary of standard deviation (std) and speckle signal-to-noise ratio (sSNR) evaluated using the envelope signals in the hyperechoic, anechoic and background regions imaged with 1-view and 8-view acquisitions. The hyperechoic, anechoic and background regions are defined in Fig. 4A.

	std		sSNR	
	1-view	8-view	1-view	8-view
Hyperechoic	5.74	5.62	1.78	1.89
Anechoic	6.07	5.91	1.42	1.69
Background	29.48	5.77	1.16	1.79

Table S5.

Quantitative analysis on a contrast phantom with different reconstruction algorithms. Summary of contrast ratio (CR) and generalized contrast-to-noise ratio (gCNR) evaluated using the envelope signals in the hyperechoic, anechoic and background regions reconstructed by DAS, DAS with the original CF, and DAS with the averaged CF weights. The hyperechoic, anechoic and background regions are defined in Fig. 4A. DAS, delay-and-sum; origCF, original coherence factor; avgCF, averaged coherence factor.

	CR (dB)			gCNR		
	DAS	DAS +origCF	DAS +avgCF	DAS	DAS +origCF	DAS +avgCF
Hyperechoic	5.55	8.47	9.52	0.43	0.52	0.56
Anechoic	27.85	53.27	56.99	0.97	1.00	1.00

Movie S1. (separate file)

Comparison between 1-view versus 8-view during real-time *in vivo* human imaging with the ultrasound tomography system. The volunteer moved the left fingers and hand in the water tank. The ROI and pixel size were 100 mm × 100 mm and 0.3 mm × 0.3 mm, respectively. For 1-view, 41 plane waves (-12.5° to 12.5°) were transmitted from the array at the bottom of the images. For 8-view, 5 plane waves (-12.5° to 12.5°) were transmitted for each view and the active arrays were rotated, then the data from all 8 views (40 plane waves total) were coherently compounded.

Movie S2. (separate file)

Comparison between 1-view versus 8-view during real-time *in vivo* human imaging with the ultrasound tomography system. The volunteer moved the left fingers and hand in the water tank. The ROI and pixel size were 100 mm × 100 mm and 0.3 mm × 0.3 mm, respectively. For 1-view, 73 plane waves (-12.5° to 12.5°) were transmitted from the array at the bottom of the images. For 8-view, 9 plane waves (-12.5° to 12.5°) were transmitted for each view and the active arrays were rotated, then the data from all 8 views (72 plane waves total) were coherently compounded.

Movie S3. (separate file)

Real-time *in vivo* human imaging with the ultrasound tomography system. The volunteer moved the right hand and forearm up and down in the water tank. The region of interest (ROI) and pixel size were 100 × 100 mm and 0.3 × 0.3 mm, respectively. 5 plane waves (-12.5° to 12.5°) were transmitted for each view (400 plane waves) and the active arrays were rotated, then the data from all 8 views were coherently compounded. The effective frame rate was ~25 Hz and dynamic range was set to 65 dB.
